# 4-[Amino­(3-methyl­phen­yl)methyl­idene]-2-(3-methyl­phen­yl)-1*H*-imidazol-5(4*H*)-one ethanol hemisolvate

**DOI:** 10.1107/S160053681205163X

**Published:** 2013-01-04

**Authors:** M. Prabhuswamy, S. Madan Kumar, C. P. Muneer, P. M. Shafi, N. K. Lokanath

**Affiliations:** a Department of Studies in Physics, Manasagangotri, University of Mysore, Mysore, India 570 006; b Department of Chemistry, Calicut University, Kerala, India 673635

## Abstract

In the title compound, C_18_H_17_N_3_O·0.5C_2_H_5_OH, the dihedral angles between the central imidazole rings and the pendant benzene rings are 42.06 (15) and 2.01 (16)° in one asymmetric mol­ecule and 47.91 (15) and 7.31 (14)° in the other. An intra­molecular N—H⋯O hydrogen bond occurs in each imidazole mol­ecule. In the crystal, the components are connected by O—H⋯N, N—H⋯O, C—H⋯O and N—H⋯N hydrogen bonds. Weak aromatic π–π inter­actions also occur [shortest centroid–centroid distance = 3.684 (3) Å].

## Related literature

For background to imidazoles, see: Shi *et al.* (2011 ▶). For a related structure, see: Chang *et al.* (2012 ▶). For further synthetic details, see: Shafi *et al.* (2005 ▶).
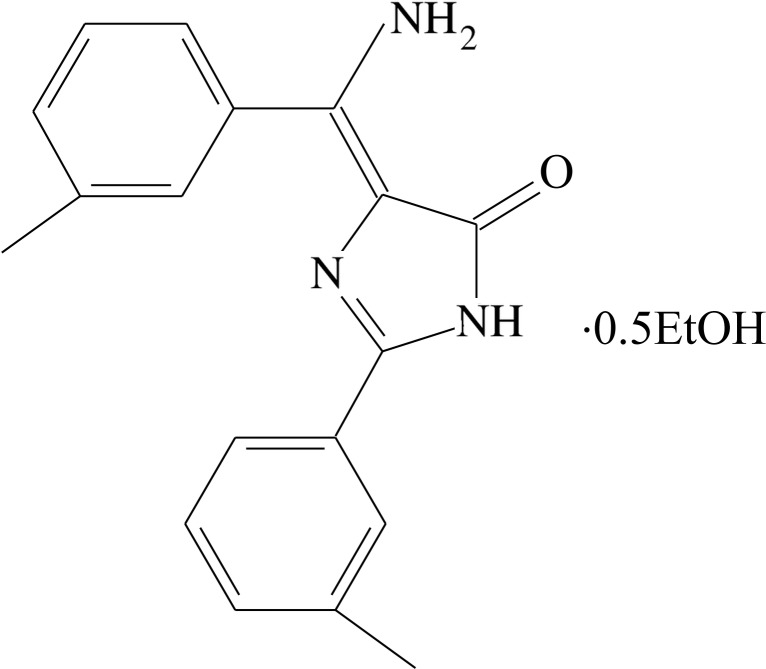


## Experimental

### 

#### Crystal data

C_18_H_17_N_3_O·0.5C_2_H_6_O
*M* *_r_* = 314.38
Triclinic, 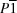

*a* = 8.227 (4) Å
*b* = 13.505 (7) Å
*c* = 16.044 (8) Å
α = 87.451 (8)°
β = 78.869 (9)°
γ = 80.241 (9)°
*V* = 1723.6 (15) Å^3^
*Z* = 4
Mo *K*α radiation
μ = 0.08 mm^−1^
*T* = 300 K
0.23 × 0.22 × 0.21 mm

#### Data collection

Oxford Diffraction Xcalibur CCD diffractometer
16691 measured reflections
6512 independent reflections
4294 reflections with *I* > 2σ(*I*)
*R*_int_ = 0.049

#### Refinement

*R*[*F* ^2^ > 2σ(*F* ^2^)] = 0.070
*wR*(*F* ^2^) = 0.227
*S* = 1.02
6512 reflections
430 parameters
H-atom parameters constrained
Δρ_max_ = 0.35 e Å^−3^
Δρ_min_ = −0.28 e Å^−3^

### 

Data collection: *CrysAlis PRO* (Oxford Diffraction, 2009 ▶); cell refinement: *CrysAlis PRO*; data reduction: *CrysAlis PRO*; program(s) used to solve structure: *SHELXS97* (Sheldrick, 2008 ▶); program(s) used to refine structure: *SHELXL97* (Sheldrick, 2008 ▶); molecular graphics: *Mercury* (Macrae *et al.*, 2006 ▶); software used to prepare material for publication: *Mercury*.

## Supplementary Material

Crystal structure: contains datablock(s) global, I. DOI: 10.1107/S160053681205163X/hb7015sup1.cif

Structure factors: contains datablock(s) I. DOI: 10.1107/S160053681205163X/hb7015Isup2.hkl

Supplementary material file. DOI: 10.1107/S160053681205163X/hb7015Isup3.cml

Additional supplementary materials:  crystallographic information; 3D view; checkCIF report

## Figures and Tables

**Table 1:** Hydrogen-bond geometry (Å, °)

*D*—H⋯*A*	*D*—H	H⋯*A*	*D*⋯*A*	*D*—H⋯*A*
N3*B*—H3*B*1⋯O1*B*	0.86	2.07	2.748 (3)	135
O2—H2⋯N1*B*	1.09	1.84	2.917 (3)	170
N3*B*—H3*B*2⋯N1*A*	0.86	2.37	3.033 (3)	134
N2*A*—H2*A*⋯O1*A* ^i^	0.86	1.95	2.803 (3)	170
N2*B*—H2*B*⋯O1*B* ^ii^	0.86	1.97	2.805 (3)	164
N3*A*—H3*A*1⋯O1*A*	0.86	2.12	2.783 (3)	134
N3*A*—H3*A*2⋯O2^iii^	0.86	2.02	2.857 (3)	163
C12*A*—H12*A*⋯O1*A* ^i^	0.93	2.59	3.492 (4)	164
C12*B*—H12*B*⋯O1*B* ^ii^	0.93	2.54	3.455 (3)	168
C16*B*—H16*B*⋯O2	0.93	2.37	3.284 (4)	168

Symmetry codes: (i) 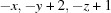; (ii) 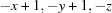; (iii) 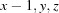.

**Fig. 1.**
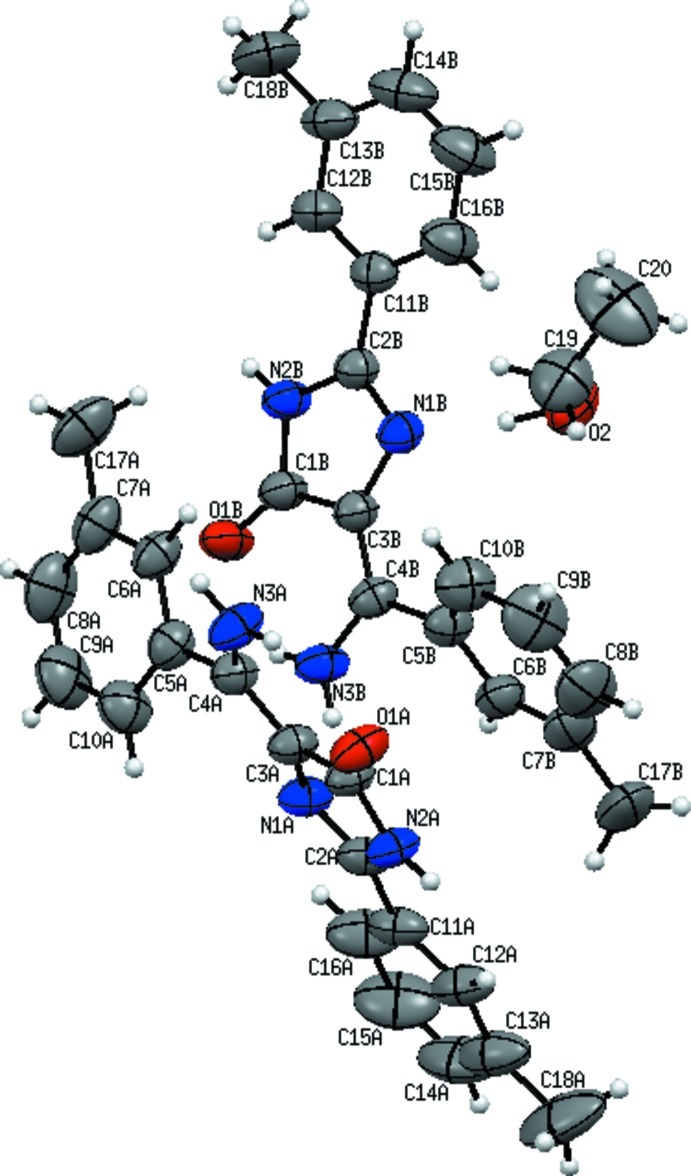
ORTEP diagram of the title compound with 50% probability ellipsoids.

**Fig. 2.**
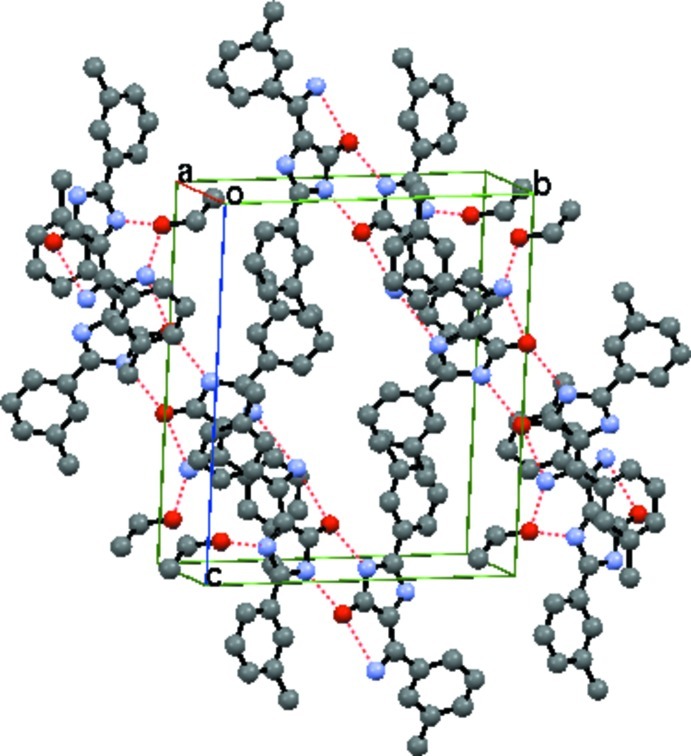
Packing diagram of the title compound, viewed along the crystallographic a axis.

## supplementary crystallographic information

### Comment

Imidazole derivatives exhibits various bioactivities (Shi *et al.*, 2011). As part of our studies in the area, we now report the structure of the title solvate, (I).

The asymmetric unit consists of two symmetry-independent molecules (*A* and *B*) of the title compound and the solvent molecule (ethanol) as shown in Fig. 1. The conformations of the two molecules are almost same which is confirmed by the dihedral angles [42.72 (14)° and 41.46 (15)° between aromatic rings of molecule A and molecule B, respectively]. The geometry of the compound is similar to (*Z*)-4-(2-Hydroxybenzylidene)-1-methyl-2-phenyl-1*H*-imidazol-5(4*H*)-one (Chang *et al.*, 2012).

The molecules are connected by hydrogen bonds of the type N—H···O, C—H···O and N—H···N (Fig. 2.). Table 1 shows the geometry of intramolecular and intermolecular hydrogen bond. In addition, weak interactions *π—π* occur [shortest centroid-centroid distance = 3.684 (3) Å].

### Experimental

The title molecule was synthesized according to the reported procedure (Shafi *et al.*, 2005) and yellow blocks were recrystallized from ethanol solution.

### Refinement

All the hydrogen atoms of the compound are fixed geometrically (N—H = 0.86 and C—H= 0.93–0.97 Å) and allowed to ride on their parent atoms. The H2 atom on O2 atom is located in a difference Fourier map and isotropically refined.

### Crystal data

**Table:**

C_18_H_17_N_3_O·0.5C_2_H_6_O	*Z* = 4
*M**_r_* = 314.38	*F*(000) = 668
Triclinic, *P*1	*D*_x_ = 1.212 Mg m^−^^3^
Hall symbol: -P 1	Mo *K*α radiation, λ = 0.71073 Å
*a* = 8.227 (4) Å	Cell parameters from 6512 reflections
*b* = 13.505 (7) Å	θ = 1.3–25.7°
*c* = 16.044 (8) Å	µ = 0.08 mm^−^^1^
α = 87.451 (8)°	*T* = 300 K
β = 78.869 (9)°	Block, yellow
γ = 80.241 (9)°	0.23 × 0.22 × 0.21 mm
*V* = 1723.6 (15) Å^3^	

### Data collection

**Table:**

Oxford Diffraction Xcalibur CCD diffractometer	4294 reflections with *I* > 2σ(*I*)
Radiation source: fine-focus sealed tube	*R*_int_ = 0.049
Graphite monochromator	θ_max_ = 25.7°, θ_min_ = 1.3°
Detector resolution: 16.0839 pixels mm^-1^	*h* = −10→10
ω scans	*k* = −16→16
16691 measured reflections	*l* = −19→19
6512 independent reflections	

### Refinement

**Table:**

Refinement on *F*^2^	Primary atom site location: structure-invariant direct methods
Least-squares matrix: full	Secondary atom site location: difference Fourier map
*R*[*F*^2^ > 2σ(*F*^2^)] = 0.070	Hydrogen site location: inferred from neighbouring sites
*wR*(*F*^2^) = 0.227	H-atom parameters constrained
*S* = 1.02	*w* = 1/[σ^2^(*F*_o_^2^) + (0.1503*P*)^2^] where *P* = (*F*_o_^2^ + 2*F*_c_^2^)/3
6512 reflections	(Δ/σ)_max_ < 0.001
430 parameters	Δρ_max_ = 0.35 e Å^−^^3^
0 restraints	Δρ_min_ = −0.28 e Å^−^^3^

### Special details

**Table:**

Geometry. Bond distances, angles *etc*. have been calculated using the rounded fractional coordinates. All su's are estimated from the variances of the (full) variance-covariance matrix. The cell e.s.d.'s are taken into account in the estimation of distances, angles and torsion angles
Refinement. Refinement on *F*^2^ for ALL reflections except those flagged by the user for potential systematic errors. Weighted *R*-factors *wR* and all goodnesses of fit *S* are based on *F*^2^, conventional *R*-factors *R* are based on *F*, with *F* set to zero for negative *F*^2^. The observed criterion of *F*^2^ > σ(*F*^2^) is used only for calculating -*R*-factor-obs *etc*. and is not relevant to the choice of reflections for refinement. *R*-factors based on *F*^2^ are statistically about twice as large as those based on *F*, and *R*-factors based on ALL data will be even larger.

### Fractional atomic coordinates and isotropic or equivalent isotropic displacement parameters (Å^2^)

**Table:**

	*x*	*y*	*z*	*U*_iso_*/*U*_eq_	
O1A	−0.0441 (2)	0.99511 (11)	0.39005 (10)	0.0627 (6)	
N1A	0.1176 (3)	0.73476 (13)	0.39638 (11)	0.0513 (6)	
N2A	0.0766 (3)	0.87156 (13)	0.47653 (11)	0.0512 (6)	
N3A	−0.0639 (3)	0.90481 (13)	0.24056 (12)	0.0605 (8)	
C1A	0.0191 (3)	0.90522 (15)	0.40432 (13)	0.0483 (7)	
C2A	0.1360 (3)	0.76943 (16)	0.46836 (13)	0.0491 (8)	
C3A	0.0423 (3)	0.81787 (15)	0.35360 (13)	0.0474 (7)	
C4A	−0.0054 (3)	0.81766 (16)	0.27501 (14)	0.0494 (8)	
C5A	0.0080 (3)	0.72533 (16)	0.22539 (15)	0.0534 (8)	
C6A	0.0597 (3)	0.72778 (17)	0.13764 (15)	0.0561 (9)	
C7A	0.0713 (4)	0.64447 (19)	0.08829 (17)	0.0655 (10)	
C8A	0.0268 (4)	0.5581 (2)	0.1294 (2)	0.0849 (15)	
C9A	−0.0246 (4)	0.5539 (2)	0.2159 (2)	0.0839 (13)	
C10A	−0.0332 (4)	0.63688 (18)	0.26547 (18)	0.0681 (10)	
C11A	0.2082 (3)	0.70925 (17)	0.53471 (14)	0.0568 (8)	
C12A	0.2279 (3)	0.7520 (2)	0.60806 (15)	0.0658 (9)	
C13A	0.2944 (4)	0.6950 (3)	0.67236 (18)	0.0857 (13)	
C14A	0.3427 (5)	0.5938 (3)	0.6602 (2)	0.1033 (16)	
C15A	0.3267 (5)	0.5506 (3)	0.5874 (3)	0.1066 (16)	
C16A	0.2582 (4)	0.6063 (2)	0.52386 (19)	0.0822 (13)	
C17A	0.1312 (5)	0.6476 (2)	−0.00652 (18)	0.0885 (13)	
C18A	0.3093 (6)	0.7438 (4)	0.7527 (2)	0.134 (2)	
O1B	0.4531 (2)	0.52071 (12)	0.11551 (9)	0.0627 (7)	
N1B	0.6174 (3)	0.74818 (12)	0.07227 (11)	0.0476 (6)	
N2B	0.5866 (3)	0.61392 (13)	0.00437 (11)	0.0538 (7)	
N3B	0.3968 (3)	0.62791 (13)	0.26286 (11)	0.0549 (7)	
C1B	0.5175 (3)	0.59695 (16)	0.08761 (13)	0.0489 (7)	
C2B	0.6424 (3)	0.70466 (15)	−0.00143 (13)	0.0456 (7)	
C3B	0.5359 (3)	0.68378 (15)	0.13094 (13)	0.0452 (7)	
C4B	0.4739 (3)	0.69788 (15)	0.21746 (13)	0.0453 (7)	
C5B	0.4914 (3)	0.78375 (16)	0.26738 (14)	0.0495 (8)	
C6B	0.5509 (3)	0.76373 (19)	0.34345 (14)	0.0569 (9)	
C7B	0.5657 (4)	0.8387 (2)	0.39569 (16)	0.0722 (11)	
C8B	0.5185 (5)	0.9354 (2)	0.3714 (2)	0.0912 (14)	
C9B	0.4569 (5)	0.9589 (2)	0.2971 (2)	0.0895 (15)	
C10B	0.4438 (4)	0.88250 (18)	0.24354 (18)	0.0703 (10)	
C11B	0.7173 (3)	0.74460 (16)	−0.08466 (14)	0.0493 (8)	
C12B	0.7199 (3)	0.69606 (19)	−0.15940 (14)	0.0580 (9)	
C13B	0.7846 (4)	0.7339 (2)	−0.23907 (15)	0.0660 (10)	
C14B	0.8467 (4)	0.8231 (2)	−0.24228 (18)	0.0792 (11)	
C15B	0.8488 (4)	0.8717 (2)	−0.1686 (2)	0.0849 (13)	
C16B	0.7855 (4)	0.83274 (19)	−0.08983 (17)	0.0707 (10)	
C17B	0.6310 (5)	0.8138 (3)	0.47749 (18)	0.1048 (18)	
C18B	0.7868 (5)	0.6790 (3)	−0.31937 (18)	0.1035 (15)	
O2	0.7675 (3)	0.92206 (12)	0.09939 (11)	0.0719 (7)	
C19	0.6601 (5)	1.0132 (2)	0.0840 (2)	0.0910 (13)	
C20	0.7531 (6)	1.0820 (3)	0.0319 (3)	0.139 (2)	
H2A	0.07600	0.90750	0.51960	0.0610*	
H3A1	−0.07170	0.96000	0.26690	0.0730*	
H3A2	−0.09390	0.90580	0.19200	0.0730*	
H6A	0.08730	0.78710	0.11140	0.0670*	
H8A	0.03190	0.50130	0.09760	0.1020*	
H9A	−0.05400	0.49480	0.24160	0.1000*	
H10A	−0.06580	0.63350	0.32420	0.0820*	
H12A	0.19580	0.82100	0.61490	0.0790*	
H14A	0.38680	0.55400	0.70190	0.1240*	
H15A	0.36280	0.48190	0.58010	0.1280*	
H16A	0.24620	0.57540	0.47520	0.0990*	
H17A	0.18320	0.58160	−0.02610	0.1330*	
H17B	0.21130	0.69280	−0.02010	0.1330*	
H17C	0.03730	0.67060	−0.03380	0.1330*	
H18A	0.20380	0.78420	0.77580	0.2000*	
H18B	0.39480	0.78550	0.74000	0.2000*	
H18C	0.33860	0.69280	0.79330	0.2000*	
H3B1	0.38570	0.57460	0.23860	0.0660*	
H3B2	0.35840	0.63630	0.31630	0.0660*	
H2B	0.59370	0.57440	−0.03720	0.0650*	
H6B	0.58170	0.69720	0.35940	0.0680*	
H8B	0.52790	0.98730	0.40550	0.1090*	
H9B	0.42400	1.02580	0.28270	0.1070*	
H10B	0.40410	0.89780	0.19320	0.0850*	
H12B	0.67710	0.63620	−0.15630	0.0700*	
H14B	0.88770	0.85070	−0.29470	0.0950*	
H15B	0.89310	0.93100	−0.17180	0.1020*	
H16B	0.78850	0.86550	−0.04050	0.0850*	
H17D	0.67160	0.87080	0.49460	0.1570*	
H17E	0.72090	0.75760	0.46860	0.1570*	
H17F	0.54190	0.79710	0.52110	0.1570*	
H18D	0.70340	0.71490	−0.34890	0.1560*	
H18E	0.76260	0.61260	−0.30510	0.1560*	
H18F	0.89560	0.67470	−0.35510	0.1560*	
H2	0.70060	0.85980	0.09520	0.100 (10)*	
H19A	0.60600	1.04450	0.13770	0.1090*	
H19B	0.57320	0.99790	0.05580	0.1090*	
H20A	0.82880	1.10430	0.06280	0.2090*	
H20B	0.67660	1.13890	0.01690	0.2090*	
H20C	0.81590	1.04880	−0.01890	0.2090*	

### Atomic displacement parameters (Å^2^)

**Table:**

	*U*^11^	*U*^22^	*U*^33^	*U*^12^	*U*^13^	*U*^23^
O1A	0.1029 (15)	0.0380 (8)	0.0485 (9)	0.0014 (8)	−0.0286 (9)	−0.0005 (7)
N1A	0.0665 (14)	0.0413 (9)	0.0399 (10)	0.0003 (9)	−0.0033 (9)	0.0015 (8)
N2A	0.0741 (14)	0.0424 (10)	0.0350 (9)	−0.0013 (9)	−0.0125 (9)	0.0011 (7)
N3A	0.0969 (17)	0.0400 (10)	0.0477 (11)	−0.0039 (10)	−0.0271 (11)	−0.0022 (8)
C1A	0.0656 (16)	0.0411 (11)	0.0374 (11)	−0.0052 (10)	−0.0120 (10)	0.0032 (9)
C2A	0.0575 (16)	0.0434 (11)	0.0383 (12)	0.0016 (10)	0.0018 (10)	0.0026 (9)
C3A	0.0608 (16)	0.0401 (11)	0.0385 (11)	−0.0029 (10)	−0.0073 (10)	0.0009 (9)
C4A	0.0617 (16)	0.0419 (11)	0.0436 (12)	−0.0087 (10)	−0.0076 (11)	−0.0009 (9)
C5A	0.0607 (17)	0.0447 (12)	0.0555 (14)	−0.0047 (11)	−0.0149 (12)	−0.0060 (10)
C6A	0.0714 (18)	0.0470 (12)	0.0521 (14)	−0.0044 (11)	−0.0204 (12)	−0.0060 (10)
C7A	0.0744 (19)	0.0567 (15)	0.0693 (17)	−0.0011 (13)	−0.0275 (14)	−0.0178 (12)
C8A	0.101 (3)	0.0562 (16)	0.104 (3)	−0.0092 (15)	−0.032 (2)	−0.0304 (16)
C9A	0.102 (3)	0.0511 (15)	0.103 (2)	−0.0284 (15)	−0.016 (2)	−0.0026 (15)
C10A	0.080 (2)	0.0510 (14)	0.0725 (18)	−0.0167 (13)	−0.0069 (14)	−0.0009 (12)
C11A	0.0572 (16)	0.0570 (13)	0.0443 (13)	0.0059 (11)	0.0032 (11)	0.0131 (10)
C12A	0.0644 (18)	0.0803 (17)	0.0419 (13)	0.0123 (13)	−0.0066 (12)	0.0088 (12)
C13A	0.061 (2)	0.127 (3)	0.0538 (17)	0.0172 (18)	−0.0069 (14)	0.0219 (17)
C14A	0.090 (3)	0.122 (3)	0.075 (2)	0.021 (2)	−0.0047 (18)	0.050 (2)
C15A	0.129 (3)	0.072 (2)	0.096 (3)	0.0235 (19)	−0.008 (2)	0.0330 (19)
C16A	0.110 (3)	0.0532 (15)	0.0663 (17)	0.0128 (15)	−0.0003 (16)	0.0127 (13)
C17A	0.118 (3)	0.0807 (19)	0.0674 (19)	0.0051 (19)	−0.0315 (19)	−0.0282 (15)
C18A	0.124 (4)	0.204 (5)	0.063 (2)	0.032 (3)	−0.046 (2)	0.006 (3)
O1B	0.0975 (15)	0.0513 (9)	0.0395 (9)	−0.0283 (9)	0.0016 (8)	−0.0036 (7)
N1B	0.0592 (13)	0.0415 (9)	0.0422 (10)	−0.0075 (8)	−0.0107 (9)	−0.0008 (7)
N2B	0.0795 (15)	0.0466 (10)	0.0353 (10)	−0.0181 (10)	−0.0031 (9)	−0.0044 (7)
N3B	0.0820 (16)	0.0425 (10)	0.0365 (10)	−0.0093 (10)	−0.0023 (9)	−0.0032 (8)
C1B	0.0652 (16)	0.0449 (11)	0.0358 (11)	−0.0106 (11)	−0.0061 (10)	−0.0012 (9)
C2B	0.0515 (15)	0.0433 (11)	0.0414 (12)	−0.0081 (10)	−0.0072 (10)	0.0010 (9)
C3B	0.0567 (15)	0.0409 (11)	0.0384 (11)	−0.0097 (10)	−0.0087 (10)	0.0003 (8)
C4B	0.0549 (15)	0.0401 (10)	0.0392 (11)	0.0011 (10)	−0.0119 (10)	−0.0017 (9)
C5B	0.0564 (16)	0.0433 (11)	0.0451 (12)	−0.0024 (10)	−0.0038 (10)	−0.0074 (9)
C6B	0.0665 (18)	0.0626 (14)	0.0410 (13)	−0.0128 (12)	−0.0053 (11)	−0.0061 (10)
C7B	0.083 (2)	0.085 (2)	0.0476 (15)	−0.0267 (16)	0.0057 (13)	−0.0210 (13)
C8B	0.117 (3)	0.082 (2)	0.073 (2)	−0.0251 (19)	0.0020 (19)	−0.0369 (17)
C9B	0.110 (3)	0.0435 (14)	0.106 (3)	−0.0006 (15)	−0.004 (2)	−0.0215 (15)
C10B	0.088 (2)	0.0490 (13)	0.0680 (17)	−0.0001 (13)	−0.0087 (15)	−0.0048 (12)
C11B	0.0511 (15)	0.0500 (12)	0.0452 (12)	−0.0055 (10)	−0.0085 (10)	0.0043 (9)
C12B	0.0640 (17)	0.0655 (14)	0.0451 (13)	−0.0166 (12)	−0.0081 (11)	0.0062 (11)
C13B	0.0658 (19)	0.0851 (18)	0.0425 (13)	−0.0078 (14)	−0.0058 (12)	0.0111 (12)
C14B	0.082 (2)	0.085 (2)	0.0573 (17)	−0.0067 (16)	0.0083 (14)	0.0228 (15)
C15B	0.102 (3)	0.0668 (17)	0.078 (2)	−0.0282 (17)	0.0131 (17)	0.0088 (15)
C16B	0.093 (2)	0.0562 (14)	0.0600 (16)	−0.0221 (14)	0.0012 (14)	0.0013 (12)
C17B	0.113 (3)	0.165 (4)	0.0477 (17)	−0.052 (3)	−0.0131 (17)	−0.0209 (19)
C18B	0.121 (3)	0.147 (3)	0.0449 (16)	−0.040 (3)	−0.0064 (17)	0.0003 (18)
O2	0.1014 (15)	0.0543 (10)	0.0702 (12)	−0.0129 (10)	−0.0422 (11)	0.0041 (8)
C19	0.110 (3)	0.0644 (17)	0.101 (2)	−0.0073 (17)	−0.034 (2)	0.0074 (16)
C20	0.144 (4)	0.073 (2)	0.189 (5)	−0.012 (2)	−0.019 (3)	0.047 (3)

### Geometric parameters (Å, º)

**Table:**

O1A—C1A	1.266 (3)	C16A—H16A	0.9300
O1B—C1B	1.262 (3)	C17A—H17B	0.9600
O2—C19	1.430 (4)	C17A—H17C	0.9600
O2—H2	1.0900	C17A—H17A	0.9600
N1A—C3A	1.405 (3)	C18A—H18C	0.9600
N1A—C2A	1.310 (3)	C18A—H18B	0.9600
N2A—C2A	1.386 (3)	C18A—H18A	0.9600
N2A—C1A	1.366 (3)	C1B—C3B	1.433 (3)
N3A—C4A	1.332 (3)	C2B—C11B	1.478 (3)
N2A—H2A	0.8600	C3B—C4B	1.393 (3)
N3A—H3A2	0.8600	C4B—C5B	1.480 (3)
N3A—H3A1	0.8600	C5B—C6B	1.400 (3)
N1B—C3B	1.405 (3)	C5B—C10B	1.383 (3)
N1B—C2B	1.309 (3)	C6B—C7B	1.378 (4)
N2B—C2B	1.373 (3)	C7B—C8B	1.360 (4)
N2B—C1B	1.372 (3)	C7B—C17B	1.515 (4)
N3B—C4B	1.338 (3)	C8B—C9B	1.387 (5)
N2B—H2B	0.8600	C9B—C10B	1.403 (4)
N3B—H3B1	0.8600	C11B—C16B	1.392 (4)
N3B—H3B2	0.8600	C11B—C12B	1.387 (3)
C1A—C3A	1.428 (3)	C12B—C13B	1.396 (3)
C2A—C11A	1.466 (3)	C13B—C18B	1.510 (4)
C3A—C4A	1.391 (3)	C13B—C14B	1.381 (4)
C4A—C5A	1.486 (3)	C14B—C15B	1.382 (4)
C5A—C10A	1.393 (3)	C15B—C16B	1.387 (4)
C5A—C6A	1.391 (3)	C6B—H6B	0.9300
C6A—C7A	1.384 (4)	C8B—H8B	0.9300
C7A—C17A	1.507 (4)	C9B—H9B	0.9300
C7A—C8A	1.383 (4)	C10B—H10B	0.9300
C8A—C9A	1.372 (5)	C12B—H12B	0.9300
C9A—C10A	1.387 (4)	C14B—H14B	0.9300
C11A—C16A	1.390 (4)	C15B—H15B	0.9300
C11A—C12A	1.382 (3)	C16B—H16B	0.9300
C12A—C13A	1.400 (4)	C17B—H17F	0.9600
C13A—C14A	1.369 (6)	C17B—H17D	0.9600
C13A—C18A	1.508 (5)	C17B—H17E	0.9600
C14A—C15A	1.367 (6)	C18B—H18E	0.9600
C15A—C16A	1.390 (5)	C18B—H18D	0.9600
C6A—H6A	0.9300	C18B—H18F	0.9600
C8A—H8A	0.9300	C19—C20	1.440 (6)
C9A—H9A	0.9300	C19—H19A	0.9700
C10A—H10A	0.9300	C19—H19B	0.9700
C12A—H12A	0.9300	C20—H20A	0.9600
C14A—H14A	0.9300	C20—H20B	0.9600
C15A—H15A	0.9300	C20—H20C	0.9600
			
C19—O2—H2	108.00	H18A—C18A—H18B	110.00
C2A—N1A—C3A	105.64 (17)	O1B—C1B—N2B	125.39 (19)
C1A—N2A—C2A	108.07 (17)	O1B—C1B—C3B	130.48 (19)
C1A—N2A—H2A	126.00	N2B—C1B—C3B	104.13 (18)
C2A—N2A—H2A	126.00	N2B—C2B—C11B	120.62 (18)
C4A—N3A—H3A1	120.00	N1B—C2B—N2B	112.56 (19)
C4A—N3A—H3A2	120.00	N1B—C2B—C11B	126.8 (2)
H3A1—N3A—H3A2	120.00	C1B—C3B—C4B	122.2 (2)
C2B—N1B—C3B	105.51 (18)	N1B—C3B—C1B	109.22 (17)
C1B—N2B—C2B	108.56 (17)	N1B—C3B—C4B	128.51 (19)
C2B—N2B—H2B	126.00	N3B—C4B—C3B	119.66 (19)
C1B—N2B—H2B	126.00	N3B—C4B—C5B	114.64 (18)
C4B—N3B—H3B2	120.00	C3B—C4B—C5B	125.7 (2)
H3B1—N3B—H3B2	120.00	C4B—C5B—C6B	118.5 (2)
C4B—N3B—H3B1	120.00	C4B—C5B—C10B	122.4 (2)
O1A—C1A—N2A	125.16 (19)	C6B—C5B—C10B	119.1 (2)
O1A—C1A—C3A	129.9 (2)	C5B—C6B—C7B	122.6 (2)
N2A—C1A—C3A	104.90 (17)	C6B—C7B—C8B	117.6 (3)
N2A—C2A—C11A	122.44 (19)	C8B—C7B—C17B	121.4 (3)
N1A—C2A—C11A	125.3 (2)	C6B—C7B—C17B	121.0 (3)
N1A—C2A—N2A	112.28 (19)	C7B—C8B—C9B	121.9 (3)
N1A—C3A—C1A	109.09 (18)	C8B—C9B—C10B	120.5 (3)
C1A—C3A—C4A	124.12 (19)	C5B—C10B—C9B	118.4 (3)
N1A—C3A—C4A	126.79 (19)	C12B—C11B—C16B	118.4 (2)
C3A—C4A—C5A	123.8 (2)	C2B—C11B—C12B	121.0 (2)
N3A—C4A—C3A	119.0 (2)	C2B—C11B—C16B	120.6 (2)
N3A—C4A—C5A	117.2 (2)	C11B—C12B—C13B	122.3 (2)
C4A—C5A—C10A	120.9 (2)	C12B—C13B—C18B	121.1 (3)
C4A—C5A—C6A	119.5 (2)	C12B—C13B—C14B	118.0 (2)
C6A—C5A—C10A	119.6 (2)	C14B—C13B—C18B	120.9 (3)
C5A—C6A—C7A	121.9 (2)	C13B—C14B—C15B	120.8 (3)
C8A—C7A—C17A	121.3 (2)	C14B—C15B—C16B	120.6 (3)
C6A—C7A—C8A	117.5 (2)	C11B—C16B—C15B	120.0 (2)
C6A—C7A—C17A	121.3 (2)	C7B—C6B—H6B	119.00
C7A—C8A—C9A	121.7 (3)	C5B—C6B—H6B	119.00
C8A—C9A—C10A	120.8 (3)	C7B—C8B—H8B	119.00
C5A—C10A—C9A	118.6 (3)	C9B—C8B—H8B	119.00
C2A—C11A—C12A	121.8 (2)	C10B—C9B—H9B	120.00
C2A—C11A—C16A	119.3 (2)	C8B—C9B—H9B	120.00
C12A—C11A—C16A	118.9 (2)	C5B—C10B—H10B	121.00
C11A—C12A—C13A	122.3 (3)	C9B—C10B—H10B	121.00
C14A—C13A—C18A	121.5 (3)	C13B—C12B—H12B	119.00
C12A—C13A—C18A	120.8 (4)	C11B—C12B—H12B	119.00
C12A—C13A—C14A	117.7 (3)	C13B—C14B—H14B	120.00
C13A—C14A—C15A	120.7 (3)	C15B—C14B—H14B	120.00
C14A—C15A—C16A	122.0 (4)	C14B—C15B—H15B	120.00
C11A—C16A—C15A	118.4 (3)	C16B—C15B—H15B	120.00
C7A—C6A—H6A	119.00	C15B—C16B—H16B	120.00
C5A—C6A—H6A	119.00	C11B—C16B—H16B	120.00
C9A—C8A—H8A	119.00	C7B—C17B—H17E	109.00
C7A—C8A—H8A	119.00	C7B—C17B—H17F	109.00
C8A—C9A—H9A	120.00	C7B—C17B—H17D	110.00
C10A—C9A—H9A	120.00	H17D—C17B—H17F	109.00
C9A—C10A—H10A	121.00	H17E—C17B—H17F	109.00
C5A—C10A—H10A	121.00	H17D—C17B—H17E	109.00
C13A—C12A—H12A	119.00	C13B—C18B—H18E	109.00
C11A—C12A—H12A	119.00	C13B—C18B—H18F	109.00
C15A—C14A—H14A	120.00	H18D—C18B—H18E	109.00
C13A—C14A—H14A	120.00	H18D—C18B—H18F	109.00
C14A—C15A—H15A	119.00	H18E—C18B—H18F	110.00
C16A—C15A—H15A	119.00	C13B—C18B—H18D	109.00
C11A—C16A—H16A	121.00	O2—C19—C20	111.2 (3)
C15A—C16A—H16A	121.00	O2—C19—H19A	109.00
C7A—C17A—H17C	109.00	O2—C19—H19B	109.00
C7A—C17A—H17B	109.00	C20—C19—H19A	109.00
H17A—C17A—H17C	109.00	C20—C19—H19B	109.00
C7A—C17A—H17A	110.00	H19A—C19—H19B	108.00
H17B—C17A—H17C	109.00	C19—C20—H20A	110.00
H17A—C17A—H17B	109.00	C19—C20—H20B	110.00
C13A—C18A—H18B	109.00	C19—C20—H20C	110.00
C13A—C18A—H18C	109.00	H20A—C20—H20B	109.00
C13A—C18A—H18A	109.00	H20A—C20—H20C	109.00
H18A—C18A—H18C	109.00	H20B—C20—H20C	109.00
H18B—C18A—H18C	110.00		
			
C3A—N1A—C2A—N2A	0.2 (3)	C12A—C11A—C16A—C15A	−0.2 (4)
C3A—N1A—C2A—C11A	179.3 (2)	C2A—C11A—C16A—C15A	179.9 (3)
C2A—N1A—C3A—C1A	0.9 (3)	C11A—C12A—C13A—C18A	−178.2 (3)
C2A—N1A—C3A—C4A	−178.4 (2)	C11A—C12A—C13A—C14A	0.9 (5)
C2A—N2A—C1A—O1A	180.0 (2)	C12A—C13A—C14A—C15A	0.3 (6)
C2A—N2A—C1A—C3A	1.7 (3)	C18A—C13A—C14A—C15A	179.3 (4)
C1A—N2A—C2A—N1A	−1.2 (3)	C13A—C14A—C15A—C16A	−1.4 (6)
C1A—N2A—C2A—C11A	179.6 (2)	C14A—C15A—C16A—C11A	1.4 (6)
C2B—N1B—C3B—C1B	1.5 (3)	O1B—C1B—C3B—N1B	179.6 (2)
C2B—N1B—C3B—C4B	−176.4 (3)	O1B—C1B—C3B—C4B	−2.4 (4)
C3B—N1B—C2B—N2B	−1.5 (3)	N2B—C1B—C3B—N1B	−0.9 (3)
C3B—N1B—C2B—C11B	177.0 (2)	N2B—C1B—C3B—C4B	177.1 (2)
C1B—N2B—C2B—N1B	1.0 (3)	N1B—C2B—C11B—C12B	−172.8 (3)
C2B—N2B—C1B—O1B	179.6 (2)	N1B—C2B—C11B—C16B	6.5 (4)
C2B—N2B—C1B—C3B	0.0 (3)	N2B—C2B—C11B—C12B	5.6 (4)
C1B—N2B—C2B—C11B	−177.6 (2)	N2B—C2B—C11B—C16B	−175.1 (3)
O1A—C1A—C3A—N1A	−179.8 (2)	N1B—C3B—C4B—N3B	178.8 (2)
O1A—C1A—C3A—C4A	−0.5 (4)	N1B—C3B—C4B—C5B	−4.1 (4)
N2A—C1A—C3A—C4A	177.7 (2)	C1B—C3B—C4B—N3B	1.2 (4)
N2A—C1A—C3A—N1A	−1.6 (3)	C1B—C3B—C4B—C5B	178.3 (2)
N1A—C2A—C11A—C12A	178.1 (3)	N3B—C4B—C5B—C6B	47.2 (3)
N2A—C2A—C11A—C16A	177.1 (3)	N3B—C4B—C5B—C10B	−129.7 (3)
N1A—C2A—C11A—C16A	−2.0 (4)	C3B—C4B—C5B—C6B	−130.0 (3)
N2A—C2A—C11A—C12A	−2.9 (4)	C3B—C4B—C5B—C10B	53.1 (4)
C1A—C3A—C4A—C5A	−175.5 (2)	C4B—C5B—C6B—C7B	−177.6 (3)
N1A—C3A—C4A—N3A	−175.0 (2)	C10B—C5B—C6B—C7B	−0.6 (4)
N1A—C3A—C4A—C5A	3.7 (4)	C4B—C5B—C10B—C9B	176.7 (3)
C1A—C3A—C4A—N3A	5.8 (4)	C6B—C5B—C10B—C9B	−0.2 (4)
N3A—C4A—C5A—C10A	−140.5 (3)	C5B—C6B—C7B—C8B	0.6 (4)
C3A—C4A—C5A—C6A	−140.3 (3)	C5B—C6B—C7B—C17B	−179.9 (3)
C3A—C4A—C5A—C10A	40.7 (4)	C6B—C7B—C8B—C9B	0.2 (5)
N3A—C4A—C5A—C6A	38.4 (3)	C17B—C7B—C8B—C9B	−179.3 (4)
C4A—C5A—C6A—C7A	−178.8 (3)	C7B—C8B—C9B—C10B	−1.0 (6)
C10A—C5A—C6A—C7A	0.2 (4)	C8B—C9B—C10B—C5B	0.9 (5)
C6A—C5A—C10A—C9A	−1.3 (4)	C2B—C11B—C12B—C13B	177.8 (3)
C4A—C5A—C10A—C9A	177.6 (3)	C16B—C11B—C12B—C13B	−1.6 (4)
C5A—C6A—C7A—C8A	1.0 (4)	C2B—C11B—C16B—C15B	−177.2 (3)
C5A—C6A—C7A—C17A	−178.6 (3)	C12B—C11B—C16B—C15B	2.1 (4)
C6A—C7A—C8A—C9A	−1.0 (5)	C11B—C12B—C13B—C14B	−0.4 (4)
C17A—C7A—C8A—C9A	178.6 (3)	C11B—C12B—C13B—C18B	179.6 (3)
C7A—C8A—C9A—C10A	−0.2 (5)	C12B—C13B—C14B—C15B	1.8 (5)
C8A—C9A—C10A—C5A	1.4 (5)	C18B—C13B—C14B—C15B	−178.2 (3)
C2A—C11A—C12A—C13A	179.0 (3)	C13B—C14B—C15B—C16B	−1.2 (5)
C16A—C11A—C12A—C13A	−0.9 (4)	C14B—C15B—C16B—C11B	−0.8 (5)

### Hydrogen-bond geometry (Å, º)

**Table:**

*D*—H···*A*	*D*—H	H···*A*	*D*···*A*	*D*—H···*A*
N3*B*—H3*B*1···O1*B*	0.86	2.07	2.748 (3)	135
O2—H2···N1*B*	1.09	1.84	2.917 (3)	170
N3*B*—H3*B*2···N1*A*	0.86	2.37	3.033 (3)	134
N2*A*—H2*A*···O1*A*^i^	0.86	1.95	2.803 (3)	170
N2*B*—H2*B*···O1*B*^ii^	0.86	1.97	2.805 (3)	164
N3*A*—H3*A*1···O1*A*	0.86	2.12	2.783 (3)	134
N3*A*—H3*A*2···O2^iii^	0.86	2.02	2.857 (3)	163
C12*A*—H12*A*···O1*A*^i^	0.93	2.59	3.492 (4)	164
C12*B*—H12*B*···O1*B*^ii^	0.93	2.54	3.455 (3)	168
C16*B*—H16*B*···O2	0.93	2.37	3.284 (4)	168

Symmetry codes: (i) −*x*, −*y*+2, −*z*+1; (ii) −*x*+1, −*y*+1, −*z*; (iii) *x*−1, *y*, *z*.
